# Gun identification from gunshot audios for secure public places using transformer learning

**DOI:** 10.1038/s41598-022-17497-1

**Published:** 2022-08-02

**Authors:** Rahul Nijhawan, Sharik Ali Ansari, Sunil Kumar, Fawaz Alassery, Sayed M. El-kenawy

**Affiliations:** 1 School of Computer Science, University of Petroleum and Energy Studies, Dehradun, Uttarakhand India; 2 California State University Dominguez Hills, Carson, USA; 3 Department of Computer Engineering, College of Computers and Information Technology, Taif University, P.O. Box 11099, Taif, 21944 Saudi Arabia; 4 Department of Communications and Electronics, Delta Higher Institute of Engineering and Technology, Mansoura, 35111 Egypt; 5 Faculty of Artificial Intelligence, Delta University for Science and Technology, Mansoura, 35712 Egypt

**Keywords:** Computational science, Computer science

## Abstract

Increased mass shootings and terrorist activities severely impact society mentally and physically. Development of real-time and cost-effective automated weapon detection systems increases a sense of safety in public. Most of the previously proposed methods were vision-based. They visually analyze the presence of a gun in a camera frame. This research focuses on gun-type (rifle, handgun, none) detection based on the audio of its shot. Mel-frequency-based audio features have been used. We compared both convolution-based and fully self-attention-based (transformers) architectures. We found transformer architecture generalizes better on audio features. Experimental results using the proposed transformer methodology on audio clips of gunshots show classification accuracy of 93.87%, with training loss and validation loss of 0.2509 and 0.1991, respectively. Based on experiments, we are convinced that our model can effectively be used as both a standalone system and in association with visual gun-detection systems for better security.

## Introduction

**Figure 1:**
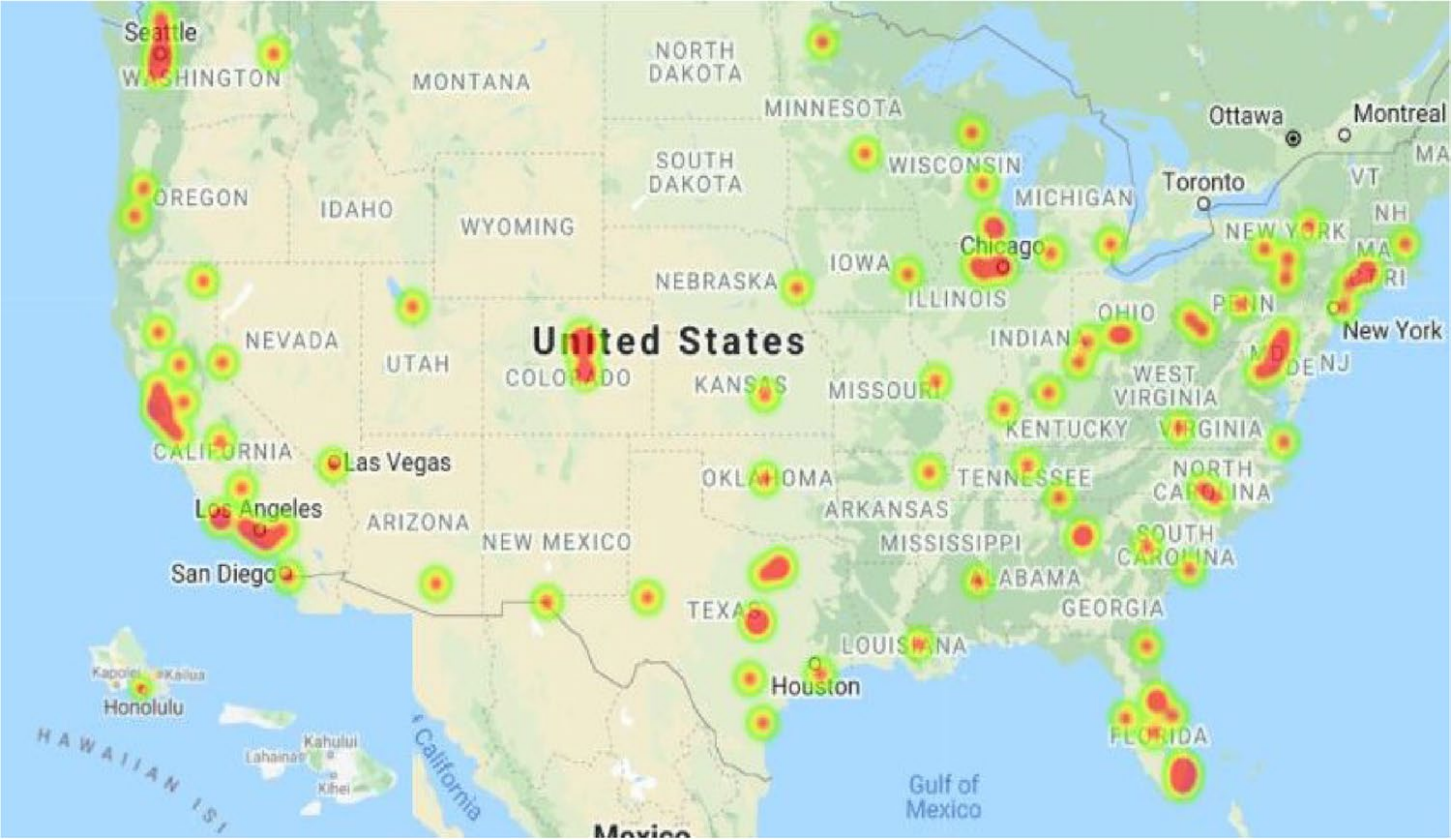
Heatmaps shown in the map represent the data collected by Mother Jones. The data shows the mass-shootings from years 1981 to 2021. More red areas means, multiple mass-shootings in that area. Software: Location History Visualizer, Source: https://locationhistoryvisualizer.com/heatmap/.

Mass shootings in public places like schools, clubs, and roads are becoming common and its rate is alarmingly increasing in the US^1^. Even the countries like Canada, Australia, France, UK have seen gun violence in public places. Addressing seriousness of problem, these countries have built unique organizations to tackle mass shootings^2^. According to Mother Jones magazine’s database, Fig. 1 shows us the public gun violence incidences across the US between 1981 and 2021. Almost all major cities get covered under the red zone. In India, Naxalite and terrorism problems are very frequent. Clearly, there should be a mechanism to detect gun violence and act fast on it irrespective of gun laws. This paper takes a step in this direction to provide a real-time and cost-effective, reliable, and robust methodology for detecting gunshots. This will help people spend quality life with family, friends, and colleagues and take immediate action if anything serious happens around them.

Guns are dangerous, especially when operated by people who want to harm others. Mass shootings in schools and public places are becoming a common place. Terrorist attacks are also increasing and creating new security challenges. Such incidents either kill or traumatize survivors for a lifetime, creating a sense of insecurity in society. This highlighted the need for a system that increases security in such situations. Even at the military level, it would help develop strategies to protect our troops at the workplace.

The computer vision community is contributing, wherever possible to protect the life of people in such uneventful situations. Automated camera-based surveillance systems are highly focused previously. These include a system to detect cold weapons, and guns, differentiating handheld items from weapons that undoubtedly increased such systems’ strength. The problem that these researchers focused on is detecting the weapon by visually seeing it on camera, preventing the mishap, and alerting the authority.

If it fails, the attack will occur, and security forces will be called to confront the attackers. At such point, forces don’t know what kind of weapons the attackers are using. By knowing their weapon, we can secure and give an edge to our security personnel. Most of the time, attackers fight from spots not covered by surveillance (including drones). At such a point, using the audio of the weapon is our only option to get information about the attacker’s weapon. The camera has limited view of capture, audio’s view is not limited. The use of audio will improve these systems by adding a small audio capture device.

Previously, researchers widely used Convolution neural network (CNN) architectures to solve computer vision problems. Although promising, CNN-based architectures have some weaknesses. Firstly, as the convolutions work with constant window size, the model helps in finding the local information rather than long-range spatial relationships between different parts of the image and the complete image^3^. Secondly, there is some loss of local information through max-pooling^4^. Inspired by Natural language processing (NLP), Vision transformers have recently been proposed as an alternative to CNNs and have shown promising results in the field of computer vision^3^. Vision transformer is free of convolutions and identifies an image as a sequence of patches, hence overcoming the issues of locality and translation invariance faced by CNN. This approach has been observed to use the hardware resources more efficiently than CNN and could be pre-trained on the public ImageNet dataset with fewer resources^3^.

In this paper, we have investigated whether we can classify different gun types from audios of their shots. The hypothesis arises as experienced military personnel can accurately tell which gun the shot is fired from by listening to audio. To test this, we created a dataset that consists of 1-s audio of shots. Transformers have proved their success from NLP to vision tasks. However, they were never used to classify audio. To replicate a human’s sense of understanding of audio, we used Mel-frequency to represent audio and developed a transformer-based classifier to predict the class of gun.

Major Challenges faced while working in this area are as follows. firstly, creating such a dataset is difficult and costly due to the legalities and risks involved. We created a dataset using audio from YouTube videos. Secondly, no such features were proposed earlier for attention-based approaches for gun audio classification. It is not known whether we can use attention directly on shot audio or if there exists some feature that can use attention to give a better result.

Our main contributions in this paper are the following:

1. We manually created a 1-s gun audio shot dataset and made it available for public use.

2.We optimized transformer hyperparameters, showing that the vision transformer has significantly improved the gunshot detection accuracy compared to the other state-of-the-art algorithms.

3. Our proposed approach has the advantage of showing sublime results on every type and aspect of the data that led to obtain unparalleled results for the task of gunshot detection when compared with state-of-the-art algorithms.

The rest of the paper is divided as: related work, the methodology that we used and dataset creation, results and discussion, conclusion, limitations and future scope.

## Related work

Choi et al. highlighted CCTV’s significance for better police service^5^. They also studied CCTV-based intelligent security systems for constructing crime-zero zone^6^. In 2018, they further analyzed the feasibility of security systems based on their economic value^7^. Adding an audio-based gun-shot detection method will enhance the reach of such systems. With a nominal increase in cost, a completely new sensory ability increases its usefulness. Liang et al.^8^ proposed a method that can detect the shooter’s position using only a few user-generated videos that include the sound of gunshots. Liang et al.^9^ in 2017 solved the issue by developing a temporal localization framework for intensive audio events in videos. The localization results are improved by the proposed method using Localized Self-Paced Reranking (LSPaR).

Morshed et al.^10^ proposed a robust neural network-based approach for audio classification. They employed an encoder that contains a sequential stack of neural networks. Further, they also used a temporal-based interpolation approach for performing the scene-level embeddings. Banoorupa et al.^11^ proposed a hybrid fingerprinting based approach for performing audio classification using LSTM. In this approach, the fingerprints were created employing the MFCC spectrum and finally converting the spectrum obtained into digital images.

Phan et al.^12^ proposed a multiclass audio classification solution for polyphonic audio event detection. They divided the event categories into multiple sets and created a multi-class problem employing a divide and conquer approach. Wang et al.^13^ proposed a 2D CNN based technique for audio classification.This algorithm was widely used in various speech recognition and classification tasks. Zhang et al.^14^ propose an AED module called Multi-Scale Time-Frequency Attention (MTFA) it informs the model where to focus along the time and frequency axes by collecting data at different resolutions, which has not been taken care in the past. Zhang et al.^15^ and Shen et al.^16^ proposed a a multiscale time-frequency convolutional recurrent neural network (MTF-CRNN) for sound event detection^15^.

Deep learning has evolved a lot over time. Earlier CNNs were being used and continue to work very well. Recently, Transformers were introduced, and state-of-the-art methods have proved their performance in both NLP and Vision tasks. In this paper, we are searching for the best way to classify guns based on their audio. We have done many experiments for this task with CNNs and transformers.

**Table 1:** This table shows various techniques employed in previous relevant audio related researches along with a research focus (purpose) and publish year.

Techniques	Purpose	Year	Accuracy (%)
Neural network, SVM, KNN, decision tree^17–20^	Audio classification	2003–2007	60.0–80.4
Multi layer perceptron^21^	Audio classification	2008	70.1
One-class SVM^22^	Audio classification	2009	76.3
Neural network^23^	Feature extraction	2010	80.0
Deep neural network^24^	Audio classification	2013	85.2–8
CNN, hierarchical neural network^25–27^	Audio Classification	2014, 2015	86.1
LSTM, RNN^28^	Audio classification	2016	88.2–89.3
CNN^29–31^	Audio tagging, deep feature extraction	2017	90.3–91.5
Deep unsupervised learning, Unsupervised learning, weakly supervised learning, attention network^32–35^	Audio event detection, audio representation, audio classification	2018	89.0–92.0
Few-shot attention, graph neural network, adversarial feature, capsule network^36–38^	Audio classification	2019	89.2–91.5
Attention-based networks, DNN ensemble^39,40^	Audio classification	2020	90.2-91.8
Attention-based networks, zero-shot federated learning^41^	Audio classification	2021	91.0-92.5
Proposed approach	Audio classification	2022	93.8

It provides a general outline of previous literature. More relevant previous works are delineated in the literature review section with important detail.

### Traditional machine learning based researches

This subsection discusses the machine learning-based audio classification approaches, and researcher’s contribution are represented in Table 1. Gunshot audio depends on various aspects, i.e., (1) firing power (size of bullet), (2) length and width of the nozzle, and (3) environment (echo). If firing power or length/width of gun nozzle changes, it reflects the weapon’s is changed. Vrysis et al.^42^ compared 1D and 2D CNNs for audio classification using various features. 2D CNN with spectrogram worked best according to them. Nanni et al^39^ used an ensemble of CNNs to classify animal sound using spectrogram and some handcrafted features. In our work, we have utilized Mel-frequency and spectrogram-based features to identify the audio sound.

### Transformer as new state of the art

Transformers are first proposed by Vaswani et al.^43^ for the text-classification task of NLP. It is complete attention based, removed the recurrent nature completely; is faster to train, and offers better performance. Experimenting with pairwise and patch-wise self-attention, Zhao et al.^44^ found both outperform CNNs. Dosovitskiy et al.^3^ used transformers for image recognition. They used a simple transformer as an encoder. Transformer architecture has proven success in different domains. Inspired by this success we employed transformers to classify audio of gun shots.

The research based on gun identification has been done by Kiktova et al.^45^ which was an extension of an intelligent acoustic event detection system. The work was based on extracting a variety of features where by using mutual information for the feature selection, the length of feature vectors were reduced. Thereafter, the Hidden Markov Model and Viterbi-based decoding algorithm utilized those obtained features. In a recent publication of 2021, Dogan^46^ presented work on predicting the gun model by identifying sound and developed an intelligent audio forensics tool. Dogan has used the fractal H-tree pattern-based classification method, where fractal and statistical features were utilized by SVM and kNN classifiers. Researchers have not explored the classification of gunshots; research has also been done on measuring and analysing the gunshot sound as they may cause hearing impairments^47^. In their study, acoustic data was collected from four different guns where sound was captured at a sampling rate of 204.8 kHz. The method developed to measure gunshot was based on using image processing techniques where Short Time Fourier Transform (STFT), was used to get the spectrogram of an audio signal. Once the spectrogram was generated, then kNN and random subspaces were used to classify them. The study found that STFT gives better accuracy than CWT. Mares and Blackburn have focused on reducing gun violence by providing an evaluation for St. Louis’ Acoustic Gunshot Detection System’s (AGDS)^48^. They have done a quasi-experimental longitudinal panel study by conducting various experiments over time. The experimental study shows a substantial increase (80%) in gunshot responses by the system. Thus, from the overall study and literature survey, it has been observed that gunshot detection plays an important role in the forensic, health sector, and security, which motivated us to work in this area.

## Methodology

### Proposed approach

**Figure 2:**
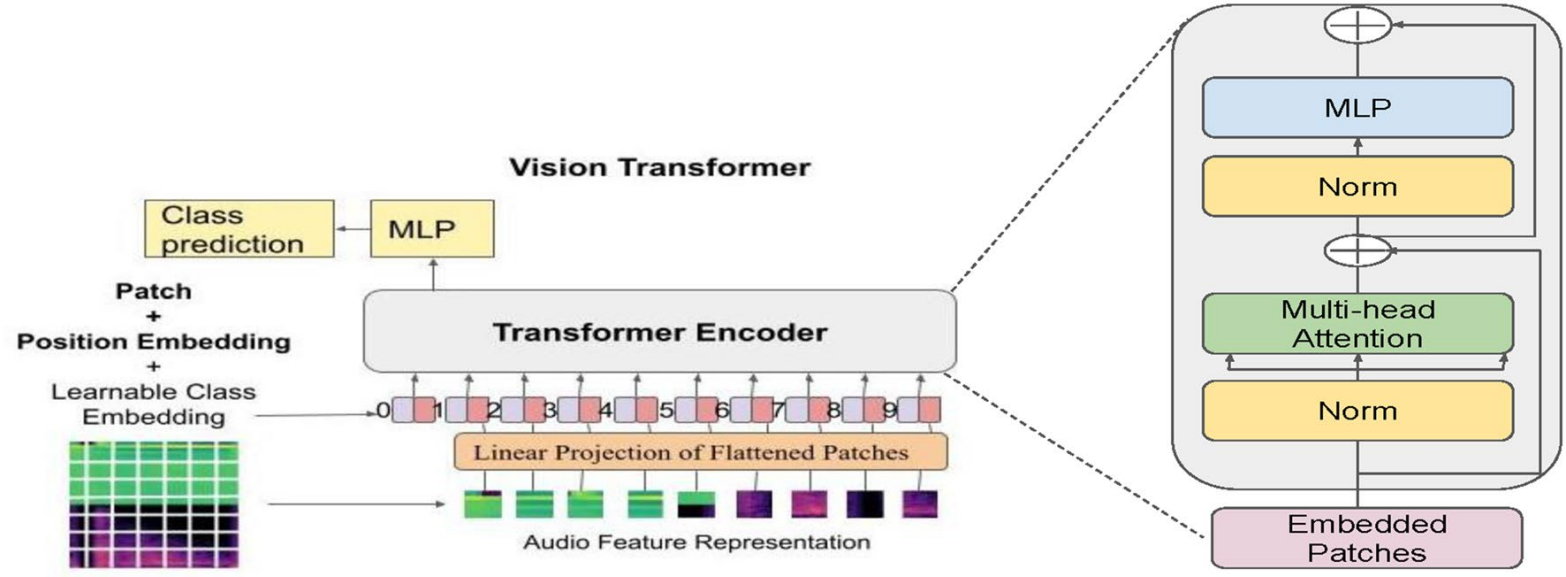
The figure shows the division of an image into patches of size $32\times 32$. The outputs from the linear projection layer is combined with positional embedding and a learnable class embedding for classification. The above diagram of the transformer encoder was derived from the work of Vaswani et al.^43^.

**Figure 3:**
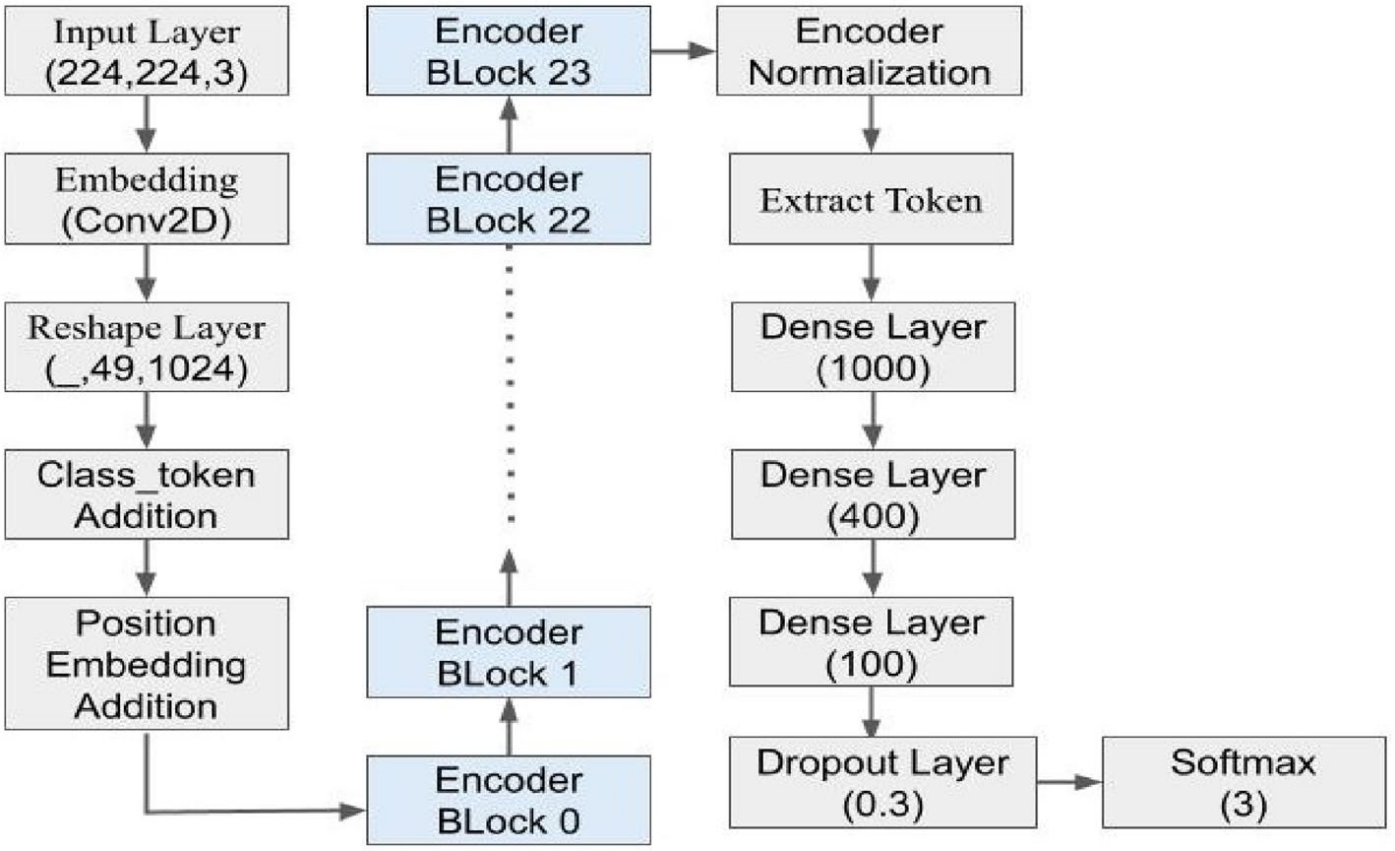
The diagram shows VIT-32 Model Architecture. It contains 24 transformer encoder blocks. The encoder block is shown in Fig. 2. in details. The arrows show the forward propagation.

In this section, the methodology which has been used to classify gunshots is discussed in detail. The proposed work approach has not been used earlier to classify audios and is thus considered to be a novelty. The approach used to carry out the work follows some steps, which are given below:

- **Loading the audio samples** To load the .*mp*3 shot samples we have used the Librosa library. This Library produces a 21,624 or 22,200 lengths *Numpy* array for a one second sample array. To make its lengths equal to nearest whole square (22,500), as deep learning algorithms work on such lengths, we padded it with another array containing ‘− 1’.
- **Extracting features from the audio:** **Mel-frequency Cepstral Coefficients (MFCCs):** Cepstral helps us to understand periodic structures in frequency which gives us information related to echos. In MFC, frequency bands are equally spaced on Mel-scale which maps audio better. MFCCs are coefficients of MFC. To obtain MFCC sequence, we have used sampling rate of 22,500 Hz, the number of output MFCC features is set to 44, length of the FFT window is 2048 samples, the number of samples between successive frames is 512, and the type2 discrete cosine transform.
- **Mel-spectogram:** A spectrogram is a visual representation of changes in frequencies of a signal with respect to time. To the human ear, the frequencies 600 Hz and 1000 Hz may sound different, but 7600 Hz and 8000 Hz sound similar. Due to non-linear transformations of the frequency scale, the pitches (frequencies) that sound less distant appear less distant on Mel-scale. In Mel-spectrogram, *y*-axis is Mel-scale, and *x*-axis is time. The Mel-spectrogram is calculated by splitting the audio signal into the windows of length 2048 samples and hop length of 512. Then, applying a fast Fourier transform to each window and separating the given audio spectrum into evenly spaced frequencies; 128 such Mel bins are taken for this purpose. Finally, for each window, based on Mel-scale frequencies, breaking down the magnitude of the audio signal into its components.
- **Vision transformer** For the image classification task, a variation is made to the traditional transformer architecture used in NLP. In our approach, the input part is processed by the encoder and the output from the encoder is fed to a Multilayer perceptron after flattening it. No decoder is used, as shown in Fig. 2. The approach treats each patch from the image like text.
  The transformer takes a linear vector with positional embedding as input. Therefore, first, 2D patches from the image are flattened to linear vector. Then, the positional embedding and class token are added to it. As shown in Fig. 3, the encoder part has many encoder blocks. Each encoder block has multiple layers of multi-headed self-attention mechanisms. The output from the encoder is normalized and sent to dense layers for image classification as shown in Fig. 3. The model is inspired from Dosovitskiy et al.^3^.
  Pre-trained model have proven their success in many researches. After identifying diminishing gradient problem, researchers have proposed using residual connections in Resnet50 paper^49^. In our experiments, while training couple of times, we found that even the Resnet50 model quickly overfits. We tried using Dropout with high value, still problem of very high difference in training and validation loss exists. As shown by^50^, transformers are better at handling such situations.
  Among various available architectures of vision transformers, we have used the L32 model for this research. The input to the transformer is a 3-channel (RGB) image of size $224 \times 224$. The image is divided into patches of shape $32\times 32$. Each patch is given as input to the linear transformation layer, which changes into a fixed-length vector. Vector of each patch has given its position information by adding a position embedding. As our problem is boiled down to image classification, a class descriptor token is also added to the patches. We pass the combined vector to the transformer encoder block. In the L32 model, we have 24 self-attention encoder blocks through which input passes for feature extraction. As our problem needs, we added two dense layers with 400 and 100 nodes, respectively. Both layers use ReLU activation. The dense layers are followed by the softmax layer, which classifies input into three classes: handgun, rifle, and none. The L32 model we choose is pre-trained on ImageNet dataset. We fine tune the model keeping each layer trainable.The Adam optimizer is used while fine-tuning with learning rate schedule of $1\times 10^{-3}$ using a warmup phase followed by a linear decay, and categorical cross-entropy is used as the loss function while training.

In this paper, we have used the concept of multi-head attention instead of single head attention by using $d_{model}$ dimentional keys, values and queries as used by Vaswani et al.^43^.

## Experimental setup

### Dataset collection and pre-processing

The dataset which has been used to carry out work contains short Gun Shot audio clips of 1 s. First, 200 videos containing multiple gunshots, were collected from YouTube. Among these 200 videos, 101 videos are of “rifle” shots, and 99 videos have “handgun” shots. The gun-shot is usually of less than 1 s, which is randomly taken audio from before and after the shot act.

To segment the 1-s shot audio, manual annotation was corrected up to a millisecond by using a video player. Thereafter, *ffmpeg* tool was used to crop annotated audio segments from videos. We kept one to several shots from each video, given they are different. In each audio clip, the background noise padding is at a different position.

To extract audio from random noise or audio containing no gun-shot, the annotations given to extract gunshot audios have been used. We have simply left the audio where the gunshots were present as these were already marked by us manually to create the dataset for the other two classes. A total of 322 audio images with noise was obtained.

Finally, all the audios were manually verified, and any unclassified audio was removed. Each audio for rifle and handgun class contains only one gunshot, which is either handgun or rifle. The dataset contains a total of 1661 audios comprising 649 handgun sample shots, 693 rifle sample shots, and 322 none or random noise in .mp3 format.

Each audio file for every class is split into set length frames in order to provide the network with sufficient and relevant data. We divided the original audio files into two categories as a first step: training samples (which constituted 70% of the original data) and test samples (which constituted 30% of the original data). This is done to prevent the network from overfitting and producing inaccurate results when tested on data that was previously used to train the network. With K = 5, we performed a K-fold cross-validation to effectively test the proposed network, as shown in Table 2.

### Comparison with other datasets and system configuration

We test our method on two additional available datasets to confirm the efficacy of the proposed method. (1) TRECVID Gunshot Videos (TREC): we have 57 videos of gunshots from the TRECVID SIN task^51^. (3) UrbanSound Gunshot Videos (Urban): from the UrbanSound dataset^52^, we have extracted 117 audio files that include gunshots. We run experiments on these two datasets, and Table 4 reports the comparison on testing accuracy for each dataset.

All the experiments were conducted inside google collaboratory. The system provided in google collaboratory has Nvidia k80 GPU with 12 GB of VRAM. The system has an Intel Xeon processor with two cores, 12 GB of RAM, and 25 GB of disk space. All the implementations were done in python 3.

## Results and discussion

This section describe the results. Table 1, shows audio classification algorithms employed in the past, with the comparison of their performance on a yearly basis. Further, we compared our proposed approach with algorithms developed by other authors, as shown in Table 4. Also, in order to check the generalizability of our proposed approach, we compared the results with two available datasets, (1) TRECVID Gunshot Videos (TREC), and (2) UrbanSound Gunshot Videos (Urban). Our proposed approach outperformed the state-of-the-art methods in all three datasets, as seen in Table 4. It is to be noted that our proposed approach produced testing accuracy of 89.0–90.0%. We could see that zero-shot federated learning produced accuracy within 83.5–86.0% (Table 4). Further, DNN Ensemble model produced accuracy of 83.0–84.5%, and capsule network produced the audio classification accuracy between 82.2 and 83.6% as shown in Table 4.

So far, CNNs^53–55^ have dominated audio classification tasks. CNN’s work is based on where it extracts significant features and edges by applying filters to a section of the data^56^. This allows the model to learn only the most important elements from the data rather than the fine details. Moreover, our proposed model works on the principle where the complete audio data is put into it, rather than only the sections that the filters can extract (or find relevant). This serves as a reason why our proposed approach outperforms the state-of-the-art approaches.

We have tried using raw audio signals directly to train Resnet50 as a baseline. When resnet50 is fine-tuned on the raw audio signal, the model overfits quickly. Training accuracy at the 50th epoch is 99.47%, while validation accuracy remained just 77.78%. On lower epochs, the validation accuracy is far poor. The training and validation loss were 0.0471 and 1.488. We found many variation in training and validation loss and classification accuracy. Then MFCC and Mel-Spectrogram features were also tested both individually and combined. When these are combined, we found that better classification accuracy is obtained. So we continued with the combined feature as our input.

**Table 2:** 5-Fold cross validation audio classification results in percentage.

Class	Accuracy	Recall	Precision	F1 score
Rifle	91.52	89.44	89.01	88.40
Handgun	90.85	89.76	88.65	89.32
Random noise	91.21	90.12	88.43	90.41
All classes	90.01	90.34	89.87	90.34

**Figure 4:**
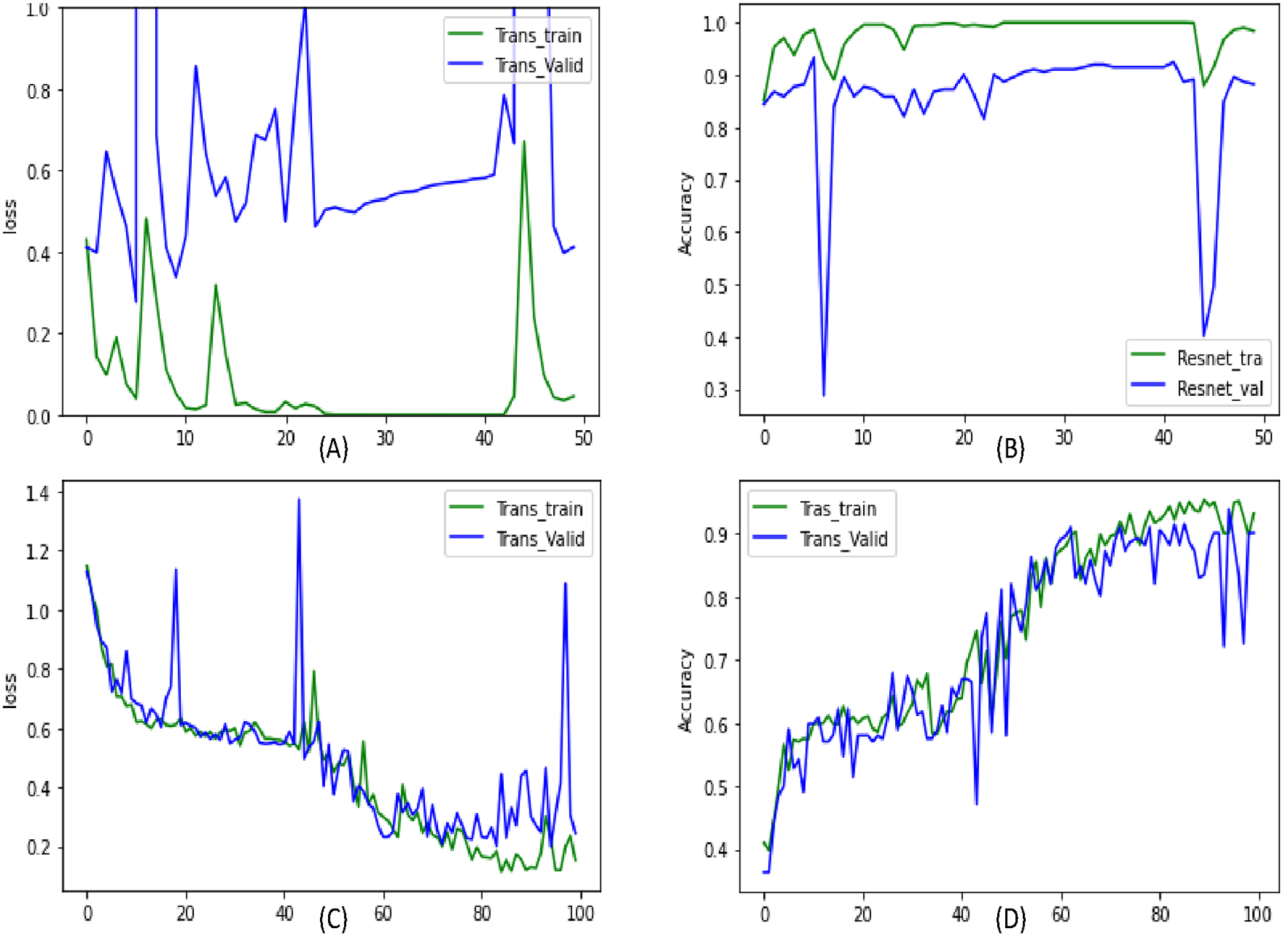
Graph (**A**) shows the training and validation accuracy curve for Resnet50 on our dataset. Graph (**B**) shows the training and validation loss curve for Resnet50 on our dataset. Graph (**C**) training and validation accuracy curve for VIT-32 on our dataset. Graph (**D**) shows the training and validation loss curve for VIT-32 on our dataset. In all the graphs, the x-axis shows the number of epochs.

We fine-tuned Resnet50 on MFCC and Mel-spectrogram features. As shown in Fig. 4, the Resnet50 model still has a lot of variations compared to the Vision transformer. The training accuracy and time for this is 98.88% and 18 h, respectively. The maximum validation classification was obtained to be 93.87% for both Resnet50 and Transformer (Table 3). However, for this accuracy vision transformer has a training loss of 0.2509 and validation loss of 0.1991. On the other side, Resnet50 has a lot of variation in Training loss $4.0\times 10^{-4}$ to 0.04 and in Validation loss of 0.2768–1.538.

The classification accuracy of vision transformer on testing dataset ranges between 89.0 and 90.0% in different training testing experiments (Table 4). Comparatively, the accuracy of Resnet50 ranges from 84.0 to 87.0% (Table 4). We split the dataset into training and testing (the terms validation and testing are interchangeably used). Our dataset size limits us to divide available audio into training testing and validation. While training, we used training data and validation data and trained the model for a fixed number of epochs. For the transformer, the best model is obtained at about 100 epochs while fine-tuning. While for resnet50 above approximately 50 epoch model start to overfit quickly.

**Table 3:** Resnet vs ViT top training accuracy and loss range in nearby epochs.

Rifle vs Handgun shot audio dataset	Top accuracy (%)	Loss range in nearby epochs
Resnet50+MFCC+MelSpectogram	93.87	0.0004–0.0400
VIT-32+MFCC+MelSpectogram	93.87	0.2768–1.538

**Table 4:** Comparison of our proposed approach with state of the art algorithms (testing accuracy range), on different available datasets.

Model	Our dataset (%)	TREC (%)	Urban (%)
Resnet50 (Baseline: raw audio)	76.0–78.0	71.0–73.0	70.0–73.4
Capsule network^37^	82.2–83.6	83.1–84.3	80.1–81.9
DNN ensemble^40^	83.0–84.5	82.2–83.4	84.0–85.0
Zero-shot federated learning^41^	83.5–86.0	82.9–24.8	83.0–85.0
Resnet50+MFCC+MelSpectogram	84.0–87.0	83.0–84.5	82.9–85.0
(PA) VT+MFCC+MelSpectogram	89.0–90.0	88.0–89.5	87.4–89.0

We trained and validated models multiple times. We performed 5-fold cross-validation as shown in Table 2.

Interestingly, the Vision Transformer, which is reputed for quick overfitting behavior, did not overfit when the MFCC+MelSpec feature in the form of an image was passed. But it overfits when raw audio is given as input. Resnet50 worked well with raw audio but overfitted when MFCC+MelSpec feature as images are passed.

While training on both raw audio and features, we observed that Resnet50 and VT created their features. Considering the recording devices, the environment (echo) was different, and background noise was present.

### Vision transformer verses CNN

Vision transformers have shown remarkable performance in several computer vision-based tasks. These architectures work on multi-head self-attention mechanisms that can accept a sequence of image patches to encode contextual cues.

We are intrigued by the fundamental differences in the operation of convolution and self-attention that have not been extensively explored in the context of robustness and generalization. It is known that convolutions learn local interactions between elements in the input domain. In contrast, self-attention has shown to learn global exchanges effectively, for example, relationships between far-off objects^57,58^. Given a query embedding, self-attention finds interactions with the other embeddings in the sequence, thereby conditioning the local content while modeling global relationships^59^. In contrast, convolutions are content-independent as the exact filter weights are applied to all inputs regardless of their distinct nature. In this paper, our analysis illustrates that Vision transformer can adjust their receptive field in order to work with the noises in the data and improve the expressivity of the representations.

## Conclusion

This paper examined the vision transformer-based approach for audio-based gun-type identification tasks. Various features like MFCC and MelSpectogram were experimented with as previous research suggested. Vision Transformer was found to work better in terms of closeness of training and validation loss, thus giving us a better fitting model. Results indicate that though only a shallow gun audio classification is done in this paper, these techniques can be employed to classify various handguns and rifles based on their shot audio.

Collecting the dataset for such a project is very difficult. It has both legal and financial issues. However, such projects are necessary.

Our dataset, though, still captured audio of gunshots in different environments; the plausible audio filters used in videos would have disturbed the original audio signal. Due to such disturbance, some critical information is missing in the audio. We felt it and therefore limited the research only to classify gun types. Had the audios been recorded using the same device with no audio filter and in various environments, we could have classified different handguns and various rifles based on shot audios. Some attackers use audio suppressors. This audio is also classifiable.

To increase the audio-based gun identification task’s, the first step will be to collect raw gun audio shots. For each gun among various types of handguns and rifles, with and without suppressors, multiple shots in different environments must be collected. As mentioned in the limitations above, this step requires the support of legal authorities and monitory support.

After dataset collection, we can train a model that will classify different types of guns based on audio of shots. Like CCTV cameras, we will attach an audio input device with CCTVs, and any gunshot will be recognized. In such a way, we can attend to such situations quickly, bypassing human intervention, which usually delays the response and causes damage to intensify.

## References

[1] Schildkraut J, Elsass HJ, Meredith K (2018). Mass shootings and the media: Why all events are not created equal. J. Crime Justice.

[2] Chalk P (2020). Domestic counter-terrorist intelligence structures in the United Kingdom, France, Canada and Australia. Stud. Conflict Terrorism.

[3] Dosovitskiy, A., Beyer, L., Kolesnikov, A., Weissenborn, D., Zhai, X., Unterthiner, T., Dehghani, M., Minderer, M., Heigold, G. & Gelly, S., et al.: An image is worth 16x16 words: Transformers for image recognition at scale. arXiv:2010.11929 (arXiv preprint) (2020).

[4] Sabour, S., Frosst, N. & Hinton, G.E. Dynamic routing between capsules. arXiv:1710.09829 (arXiv preprint) (2017).

[5] Yoo JS, Min KJ, Jeong SH, Shin DB (2016). Inter-ministerial collaboration to utilize CCTV video service operated by u-city center of South Korea. Spat. Inf. Res..

[6] Choi W-C, Na J-Y (2016). Relative importance for security systems of crime-zero zone based on spatial information. Spat. Inf. Res..

[7] Choi WC, Na JY (2018). Evaluating economic values of intelligent security services based on spatial information in South Korea. Spat. Inf. Res..

[8] Liang, J., Aronson, J. D. & Hauptmann, A.: Shooter localization using social media videos. In *Proceedings of the 27th ACM International Conference on Multimedia*, 2280–2283 (2019).

[9] Liang, J., Jiang, L. & Hauptmann, A.: Temporal localization of audio events for conflict monitoring in social media. In *2017 IEEE International Conference on Acoustics, Speech and Signal Processing (ICASSP)*, 1597–1601 (IEEE, 2017).

[10] Morshed, M.M., Ahsan, A.O., Mahmud, H. Hasan, M., et al.: Learning audio representations with mlps. arXiv:2203.08490 (arXiv preprint) (2022).

[11] Banuroopa K, Shanmuga Priyaa D (2022). Mfcc based hybrid fingerprinting method for audio classification through lstm. Int. J. Nonlinear Anal. Appl..

[12] Phan, H., Nguyen, T. N. T., Koch, P. & Mertins, A. Polyphonic audio event detection: Multi-label or multi-class multi-task classification problem?. arXiv:2201.12557 (arXiv preprint) (2022).

[13] Wang X (2022). Rainfall observation using surveillance audio. Appl. Acoust..

[14] Zhang, J., Ding, W., Kang, J. & He, L.: Multi-scale time-frequency attention for acoustic event detection. arXiv:1904.00063 (arXiv preprint) (2019).

[15] Zhang K, Cai Y, Ren Y, Ye R, He L (2020). MTF-CRNN: Multiscale time-frequency convolutional recurrent neural network for sound event detection. IEEE Access.

[16] Shen, Y.-H., He, K.-X & Zhang, W.-Q.: Learning how to listen: A temporal-frequential attention model for sound event detection. arXiv:1810.11939 (arXiv preprint) (2018).

[17] Shao, X., Xu, C. & Kankanhalli, M. S. Applying neural network on the content-based audio classification. In *Fourth International Conference on Information, Communications and Signal Processing, 2003 and the Fourth Pacific Rim Conference on Multimedia. Proceedings of the 2003 Joint*, vol. 3, 1821–1825 (IEEE, 2003).

[18] Mitra, V. & Wang, C. J. A neural network based audio content classification. In *2007 International Joint Conference on Neural Networks*, 1494–1499 (IEEE, 2007).

[19] Chen, L., Gunduz, S. & Ozsu, M. T. Mixed type audio classification with support vector machine. In *2006 IEEE International Conference on Multimedia and Expo*, 781–784 (IEEE, 2006).

[20] Zhu, Y., Ming, Z. & Huang, Q. Svm-based audio classification for content-based multimedia retrieval. In *International Workshop on Multimedia Content Analysis and Mining*, 474–482 (Springer, 2007).

[21] Mitra Vikramjit, Wang Chia-Jiu (2008). Content based audio classification: A neural network approach. Soft Comput..

[22] Jingbin Y, Shi W, Kheidorov I (2009). Audio classification based on one-class svm. J. Comput. Appl..

[23] Li TL, Chan AB, Chun A (2010). Automatic musical pattern feature extraction using convolutional neural network. Genre.

[24] Kons Z, Toledo-Ronen O, Carmel M (2013). Audio event classification using deep neural networks. Interspeech.

[25] Dieleman, S. & Schrauwen, B. End-to-end learning for music audio. In *2014 IEEE International Conference on Acoustics, Speech and Signal Processing (ICASSP)*, 6964–6968 (IEEE, 2014).

[26] Ravanelli, M., Elizalde, B., Ni, K. & Friedland, G. Audio concept classification with hierarchical deep neural networks. In *2014 22nd European Signal Processing Conference (EUSIPCO)*, 606–610 (IEEE, 2014).

[27] Piczak, K. J.: Environmental sound classification with convolutional neural networks. In *2015 IEEE 25th International Workshop on Machine Learning for Signal Processing (MLSP)*, 1–6 (IEEE, 2015).

[28] Dai, J., Liang, S., Xue, W., Ni, C. & Liu, W. Long short-term memory recurrent neural network based segment features for music genre classification. In *2016 10th International Symposium on Chinese Spoken Language Processing (ISCSLP)*, 1–5 (IEEE, 2016).

[29] Freitag M, Amiriparian S, Pugachevskiy S, Cummins N, Schuller B (2017). audeep: Unsupervised learning of representations from audio with deep recurrent neural networks. J. Mach. Learn. Res..

[30] Xu Y, Huang Q, Wang W, Foster P, Sigtia S, Jackson PJ, Plumbley MD (2017). Unsupervised feature learning based on deep models for environmental audio tagging. IEEE/ACM Trans. Audio Speech Lang. Process..

[31] Oramas, S., Nieto, O., Barbieri, F. & Serra, X. Multi-label music genre classification from audio, text, and images using deep features. arXiv:1707.04916 (arXiv preprint) (2017).

[32] Morfi, V. & Stowell, D. Data-efficient weakly supervised learning for low-resource audio event detection using deep learning. arXiv:1807.06972 (arXiv preprint) (2018).

[33] Jansen, A., Plakal, M., Pandya, R., Ellis, D. P., Hershey, S., Liu, J., Moore, R. C. & Saurous, R. A. Unsupervised learning of semantic audio representations. In *2018 IEEE International Conference on Acoustics, Speech and Signal Processing (ICASSP)*, 126–130 (IEEE, 2018).

[34] Amiriparian, S., Schmitt, M., Cummins, N., Qian, K., Dong, F. & Schuller, B. Deep unsupervised representation learning for abnormal heart sound classification. In *2018 40th Annual International Conference of the IEEE Engineering in Medicine and Biology Society (EMBC)*, 4776–4779 (IEEE, 2018). 10.1109/EMBC.2018.8513102 30441416

[35] Wu Y, Mao H, Yi Z (2018). Audio classification using attention-augmented convolutional neural network. Knowl.-Based Syst..

[36] Zhang S, Qin Y, Sun K, Lin Y (2019). Few-shot audio classification with attentional graph neural networks. Interspeech.

[37] Jain, R.: Improving performance and inference on audio classification tasks using capsule networks. arXiv:1902.05069 (arXiv preprint) (2019).

[38] Gao L, Mi H, Zhu B, Feng D, Li Y, Peng Y (2019). An adversarial feature distillation method for audio classification. IEEE Access.

[39] Nanni L, Costa YM, Aguiar RL, Mangolin RB, Brahnam S, Silla CN (2020). Ensemble of convolutional neural networks to improve animal audio classification. EURASIP J Audio Speech Music Process..

[40] Lu, H., Zhang, H. & Nayak, A. A deep neural network for audio classification with a classifier attention mechanism. arXiv:2006.09815 (arXiv preprint) (2020).

[41] Gudur, G. K. & Perepu, S. K. Zero-shot federated learning with new classes for audio classification. arXiv:2106.10019 (arXiv preprint) (2021).

[42] Vrysis L, Tsipas N, Thoidis I, Dimoulas C (2020). 1d/2d deep cnns vs temporal feature integration for general audio classification. J. Audio Eng. Soc..

[43] Vaswani, A., Shazeer, N., Parmar, N., Uszkoreit, J., Jones, L., Gomez, A.N., Kaiser, Ł. & Polosukhin, I. Attention is all you need. In *Advances in Neural Information Processing Systems*, 5998–6008 (2017).

[44] Zhao, H., Jia, J. & Koltun, V. Exploring self-attention for image recognition. In *Proceedings of the IEEE/CVF Conference on Computer Vision and Pattern Recognition*, 10076–10085 (2020).

[45] Kiktova, E., Lojka, M., Pleva, M., Juhar, J. & Cizmar, A. Gun type recognition from gunshot audio recordings. In *3rd International Workshop on Biometrics and Forensics (IWBF 2015)*, 1–6 (IEEE, 2015).

[46] Dogan S (2021). A new fractal h-tree pattern based gun model identification method using gunshot audios. Appl. Acoust..

[47] Tardif, B., Lo, D. & Goubran, R. Gunshot sound measurement and analysis. In *2021 IEEE Sensors Applications Symposium (SAS)*, 1–6 (IEEE, 2021).

[48] Mares D, Blackburn E (2021). Acoustic gunshot detection systems: A quasi-experimental evaluation in St. Louis, MO. J. Exp. Criminol..

[49] He, K., Zhang, X., Ren, S. & Sun, J. Deep residual learning for image recognition. In *Proceedings of the IEEE Conference on Computer Vision and Pattern Recognition*, 770–778 (2016).

[50] Zhang, C., Zhang, M., Zhang, S., Jin, D., Zhou, Q., Cai, Z., Zhao, H., Yi, S., Liu, X. & Liu, Z. Delving deep into the generalization of vision transformers under distribution shifts. arXiv:2106.07617 (arXiv preprint) (2021).

[51] Awad, G., Fiscus, J., Joy, D., Michel, M., Smeaton, A., Kraaij, W., Eskevich, M., Aly, R., Ordelman, R., Ritter, M., et al: Trecvid 2016: Evaluating video search, video event detection, localization, and hyperlinking. In *TREC Video Retrieval Evaluation (TRECVID)* (2016).

[52] Salamon, Justin and Jacoby, Christopher and Bello, Juan Pablo: A dataset and taxonomy for urban sound research. In *Proceedings of the 22nd ACM International Conference on Multimedia*, 1041–1044 (2014).

[53] Fang Z (2021). A high-efficient hybrid physics-informed neural networks based on convolutional neural network. IEEE Trans. Neural Netw. Learn. Syst..

[54] Zheng W, Liu X, Yin L (2021). Research on image classification method based on improved multi-scale relational network. PeerJ Comput. Sci..

[55] Zuo C, Qian J, Feng S, Yin W, Li Y, Fan P, Han J, Qian K, Chen Q (2022). Deep learning in optical metrology: A review. Light Sci. Appl..

[56] Liu R, Wang X, Lu H, Wu Z, Fan Q, Li S, Jin X (2021). Sccgan: Style and characters inpainting based on cgan. Mob. Netw. Appl..

[57] Ramachandran, P., Parmar, N., Vaswani, A., Bello, I., Levskaya, A. & Shlens, J. Stand-alone self-attention in vision models. arXiv:1906.05909 (arXiv preprint) (2019).

[58] Hu, H., Zhang, Z., Xie, Z. & Lin, S. Local relation networks for image recognition. In *Proceedings of the IEEE/CVF International Conference on Computer Vision*, 3464–3473 (2019).

[59] Vaswani, A., Ramachandran, P., Srinivas, A., Parmar, N., Hechtman, B. & Shlens, J. Scaling local self-attention for parameter efficient visual backbones. In *Proceedings of the IEEE/CVF Conference on Computer Vision and Pattern Recognition*, 12894–12904 (2021).

